# Patterns of Proliferative Activity in the Colonic Crypt Determine Crypt Stability and Rates of Somatic Evolution

**DOI:** 10.1371/journal.pcbi.1003082

**Published:** 2013-06-13

**Authors:** Rui Zhao, Franziska Michor

**Affiliations:** Department of Biostatistics and Computational Biology, Dana-Farber Cancer Institute, and Department of Biostatistics, Harvard School of Public Health, Boston, Massachusetts, United States of America; ETH Zurich, Switzerland

## Abstract

Epithelial cells in the colon are arranged in cylindrical structures called crypts in which cellular proliferation and migration are tightly regulated. We hypothesized that the proliferation patterns of cells may determine the stability of crypts as well as the rates of somatic evolution towards colorectal tumorigenesis. Here, we propose a linear process model of colonic epithelial cells that explicitly takes into account the proliferation kinetics of cells as a function of cell position within the crypt. Our results indicate that proliferation kinetics has significant influence on the speed of cell movement, kinetics of mutation propagation, and sensitivity of the system to selective effects of mutated cells. We found that, of all proliferation curves tested, those with mitotic activities concentrated near the stem cell, including the actual proliferation kinetics determined in *in vivo* labeling experiments, have a greater ability of delaying the rate of mutation accumulation in colonic stem cells compared to hypothetical proliferation curves with mitotic activities focused near the top of the crypt column. Our model can be used to investigate the dynamics of proliferation and mutation accumulation in spatially arranged tissues.

## Author Summary

Mathematical and computational models have a long and rich history in enhancing our understanding of intestinal epithelial cells. A plethora of models have been proposed to describe different aspects of cellular behavior, including cell proliferation, migration, differentiation, and mutation accumulation. Here, we present a novel approach to examine the effects of proliferation kinetics on the rate of somatic evolution in a spatially arranged model of the colon. Based on our simulation results, we demonstrate that spatially determined proliferation kinetics has the ability to delay the rate of somatic evolution, and changes in proliferation patterns can significantly affect the speed of mutation accumulation. Our work highlights the importance of considering proliferation kinetics as well as the spatial organization of tissues when investigating the dynamics of cancer initiation.

## Introduction

Colorectal cancer is the third most prevalent cancer type for both men and women in the United States, accounting for 9% of all cancer deaths [1]. This large incidence can be partially attributed to the rapid cell divisions that continuously replenish the colonic epithelium, as this large amount of cell turnover increases the risk of accumulating the genetic changes leading to colorectal tumorigenesis [2]. The identity and order of genetic alterations leading to colorectal cancer have been extensively studied [3]. The gene most frequently altered in colorectal cancer is adenomatous polyposis coli (*APC*), with more than 85% of all colorectal cancer cases harboring mutations in this gene [4]. *APC*, a tumor suppressor, is a negative regulator of the β-catenin oncoprotein [5], and mutations in *APC* lead to elevated levels of β-catenin in the cytoplasm, which in turn induce changes in proliferation, differentiation, migration, adhesion, and apoptosis [6]. Germline *APC* mutation results in the familial adenomatous polyposis (FAP) syndrome, which is characterized by an early onset of colorectal cancer in almost all afflicted individuals [7]. Other frequently altered genes in colorectal cancer include *KRAS* [8], the *SMAD* genes [9], *TP53* [10], and *MYC* [11], [12]. In addition to alterations of oncogenes and tumor suppressor genes, colorectal tumors often display a mutator phenotype, which has been broadly categorized as microsatellite instability (MIN) [13] or chromosomal instability (CIN) [14]. About 15% of sporadic colorectal cancers display MIN, caused by a loss of DNA mismatch repair gene function [13]; the remaining 85% have CIN, characterized by an excessive rate of gaining or losing whole chromosomes or parts of chromosomes, at a rate of up to 10^−2^ per chromosome per cell division [14]. An important feature associated with tumors harboring CIN is the accelerated rate of loss of heterozygosity (LOH), which increases the rate of tumor suppressor gene inactivation. It is not unusual for more than half of the genes in colorectal tumor cells to display LOH [15]. More than one hundred genes associated with CIN have been identified in yeast, many of which have human homologs [16], [17].

In addition to the genetic sequence leading to colorectal cancer, the physical architecture and proliferation kinetics of epithelial cells have also been the topic of many investigations. Epithelial cells in the colon are arranged in cylindrical compartments called crypts [18]. Each crypt contains on average 2,000 cells, with about 40 cells in circumference and 80 cells in height [19]. A small number of stem cells (4–6) are located at the bottom of the crypt [20], [21]. These cells divide to produce the differentiated progenies populating the crypt. The latter cells divide and migrate upward with limited lateral movement and are eventually shed off into the lumen of the large intestine [22]. The proliferation kinetics of cells follows a complex and spatially specific pattern, with proliferating cells concentrated at the bottom half of the crypt, near the stem cells, and the upper half of the crypt consisting of non-dividing migrating cells [19], [23]. This proliferation pattern is tightly controlled, and changes in this pattern have been shown to be associated with the progression towards colorectal cancer [24]. Quantitative measurements in animal models demonstrated that the speed of the upward migration increases from 0.02 cell positions per hour per crypt column at the bottom of the crypt to approximately 1.0 cell positions per hour near the top [25]. Under normal circumstances, the entire crypt is regenerated every 2 to 7 days [2]. Overall, the human colon contains about 10^7^ crypts, thus bringing the total number of epithelial cells in the colon to about 2×10^10^ cells.

As experiments involving colonic epithelial cells remain technically challenging or infeasible in humans, several mathematical and computational models were developed to enhance our understanding of crypt kinetics and the somatic evolution leading to colorectal cancer. Early work has led to the postulation that colorectal cancer is the result of a sequential accumulation of mutations [26]. Since then, many mathematical models have been proposed to describe the accumulation of mutations leading to colorectal cancer. For instance, some investigators have addressed the effects of tissue architecture on the rate of mutation accumulation in colonic tissues. These studies include the spatially explicit models proposed by Komarova and Wang [27] to investigate the location within the crypt at which *APC* mutations arise, by Michor *et al.* to elucidate the time during tumorigenesis at which CIN arises [28], and by Buske *et al*. to investigate the changes in tissue dynamics resulting from gains or losses of specific gene functions using an agent-based model [29]. In addition, Nowak *et al.* proposed a linear process model to study the speed of somatic evolution in colonic crypts [30]. In this model, *N* cells within a crypt column are projected onto a one-dimensional grid. During each time step, a cell is selected for reproduction. A cellular division yields two daughter cells, with one daughter occupying the original position and the other daughter residing in the position immediately to the right of the original cell. All cells on the right of the dividing cell move to the right by one position in the grid, and the last cell is shed off into the lumen of the intestine. During each cell division, a mutation may occur with a certain probability; each daughter cell has a chance of 1/2 of inheriting the mutation. Compared to a well-mixed population of cells, this linear process was shown to slow down the speed of somatic evolution and to conceal the selective effects of advantageous mutants [30]. This observation suggests that the cellular architecture of multicellular tissues has the potential to delay the onset of cancer. Several other models were designed to specifically investigate crypt kinetics. Two excellent reviews by van Leewen *et al.* [31] and de Matteis *et al.* [32] provide an in-depth discussion of these studies: a two-dimensional lattice-based model [33], [34], a one-dimensional lattice-based model with an intraepithelial growth factor gradient [35], a two-dimensional lattice-free model based on Voronoi tessellation [36], and a cellular Potts model (CPM) [37]. Recently, Mirams and Fletcher presented an integrated model incorporating both proliferation kinetics and tissue architecture for investigating mutation fixation within a crypt [38]. Using the number of proliferating cells as a proxy for proliferation kinetics, they showed that the dynamics of cell division have a significant effect on the spread of mutated cells within the population.

Despite these forays, several open questions remain regarding the effects of proliferation kinetics on the rate of mutation accumulation towards colorectal cancer. To address these issues, we developed a spatially arranged stochastic model of the colonic crypt. We investigated several different proliferation kinetics curves, including one quantitatively measured using labeling indices in the normal colonic epithelium [19], [23], and their effects on the rate of somatic evolution towards colorectal tumorigenesis. This model contributes to a quantitative understanding of the initiation and progression of colorectal cancer and can be used to investigate the effects of spatial patterns on mutation accumulation.

## Methods

### The mathematical model

**Figure 1:**
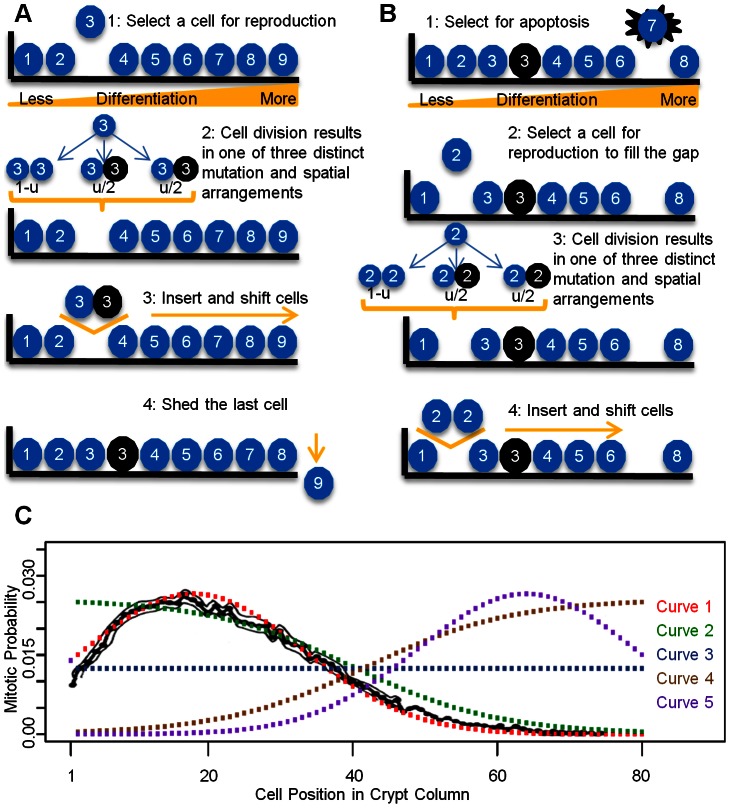
Schematics of the linear process and proliferation kinetic curves. A: We designed a linear process model to describe the essential features of cell movement in a colonic crypt. During each time step, a cell at position *i* is selected to divide. During mitosis, a mutation may occur with probability *u*, giving rise to one mutated and one wild type daughter cell, with equal probability of occupying either position *i* or *i*+1. Cell division pushes all cells to the right of position *i* upwards in the crypt by one position. The last cell is shed into the lumen of the colon. B: Cell death may occur after each round of cell division. The number of dying cells follows a Poisson distribution with mean *λ*; the positions of the dying cells are selected according to a uniform distribution. Dead cells are replaced by replenishing cell divisions. Dying cells at position *j* can only be replaced by cells of a similar (*j*+1≤*k*≤*j*+δ) or less (*k*<*j*) differentiated stage. Position *k* is selected according to the proliferation kinetics curve. If multiple cells die simultaneously, the replenishing cell divisions occur sequentially, in the order of *j_(1)_*, *j_(2)_ … j_(m)_*, where *j_(1)_*, *j_(2)_ … j_(m)_* are ordered death position. The *m* positions for the replenishing cell divisions are selected according to the reweighted kinetic curve. C: Proliferation kinetic curves as a function of cell positions. The black curve represents the measured labeling index for normal human colon using bromodeoxyuridine (BRDU) [23]. The colored curves represent the five kinetic curves under investigation. See the Methods section for details. Note that curve 1 is in good agreement with the measured labeling index.

In order to investigate the effects of proliferation kinetics on the rate of somatic evolution toward colorectal tumorigenesis, we designed a spatial model capturing the essential features of tissue architecture and cellular movement in colonic crypts. Each colonic crypt is modeled by a representative column of cells, which is projected onto a linear lattice (**Fig. 1A.**). The total number of cells per column is given by 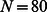, as determined by *in vivo* measurements with a measured mean of 81.9 cells (±9.7 cells) [19]. Position 1 on the left end of the lattice represents the stem cell and position 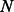 on the far right represents the apex of the crypt, close to the gut lumen. During every elementary time step of this stochastic process, a cell at position *i* is selected to divide according to a probability weight, *w_i_*, defined by a specific proliferation kinetic curve for 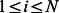, zero elsewhere. The two daughter cells are then placed into positions *i* and *i*+1, causing cells that previously resided in positions *i*+1 to 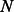 to shift by one position to the right. The last cell is shed into the gut lumen. During each division, a mutation may occur with probability *u*. If a mutation arises, then each of the daughter cells has a chance of 1/2 of inheriting the mutation. This flexible model then allows us to investigate the effects of different proliferation curves on the rate of somatic evolution. Our model closely resembles the one originally proposed by Nowak *et al.* [30], with the difference of incorporating specific proliferation kinetics.

The dynamics of proliferation is a function of the cell position in the crypt column. The proliferation kinetic curve assigns a mitotic probability to each position in the crypt column: a more proliferative position in the crypt column is represented by a larger mitotic probability. We examined five proliferation kinetic curves (**Fig. 1C.**). Position 1, the stem cell position, has the same mitotic probability in all curves, such that 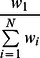 is identical for all curves. Weights for positions 2 to 80 are assigned by different proliferation curves. Proliferation curve 1 represents the measured kinetic curve, which is extrapolated from *in vivo* bromodeoxyuridine (BrdU) labeling experiments [19], [23]. The measured distribution was approximated using a normal distribution with mean 18 and standard deviation 15 to best match the 90^th^ percentile interval of the measured curve. The probability weights for choosing a cell at each position in the crypt column, *i = *2 to 80, are specified by the probability density function, 
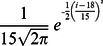
. Curve 2 represents the logistic proliferation curve generated from 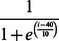; this curve is used to investigate the effects of spreading the proliferation activities upwards in the crypt column. Curve 3 represents a uniform curve. Curve 4 represents the mirror image of curve 2, with a vertical plane of reflection between positions 40 and 41. Finally, curve 5 represents the mirror image of curve 1, with a vertical plane of reflection between positions 40 and 41. Curves 4 and 5 were selected to examine the effects of proliferating activities concentrated far away from the stem cell.

In addition to the normal shedding of the last cell in the crypt column, accidental premature cell death may also occur (**Fig. 1B.**). The rates of apoptosis have been measured using terminal deoxynucleotidyl transferase dUTP nick end labeling (TUNEL) in normal cells, hyperplastic polyps, adenomas and carcinomas; in these cases, the percentage of cells being labeled ranged from 1% to 4% [39]. These observations suggest that multiple cell deaths may occur during each round of cell division. In the context of our model, such cell death may occur after each normal cell division event. The number of dying cells, *m*, follows a Poisson distribution with mean *λ*, which denotes the mean number of cell deaths per round of cell division. To incorporate the possibility of having multiple cell deaths, *λ* can vary between 0 and 3.2. The upper bound of 3.2 corresponds to 4% of the length of the crypt column, which is the observed percentage of cells undergoing apoptosis using Tunel labeling [39] . Cells are selected for death according to a particular apoptosis curve specifying the likelihood of cell death for each position in the crypt column. Due to a lack of quantitatively measured apoptosis curves as a function of cell position within the crypt column, we used the five proliferation curves discussed above as proxies for apoptosis curves and tested the resulting twenty five combinations of proliferation and apoptosis curves for their effects on the dynamics of the system.

The vacancy resulting from a cell death at position *j* can be filled by an additional cell division occurring at position *k*, where *k*≤*j*+*δ* and *δ* indicates the size of the interval in which more differentiated cells can replace the dead cell. All following numerical examples are calculated based on *δ* = 5. Position *k* is selected for a replenishing cell division according to the reweighted proliferation kinetics, 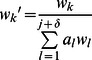, where 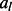 indicates the existence of a viable cell at position 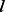. If multiple cells die simultaneously, the replenishing cell divisions occur sequentially, in the order of *j*_(1)_, *j*_(2)_ *… j*_(m)_, where *j*_(1)_, *j*_(2)_ *… j*_(*m*)_ are ordered positions for the *m* dying cells. The *m* positions for the replenishing cell divisions are again selected according to the sequentially reweighted proliferation kinetic curve. This design was chosen to ensure that the dead cell(s) can only be replaced by cells of a similar differentiation stage (*j*+1*≤k≤j*+*δ*) or a less differentiated stage (*k*<*j*). In this model, the differentiation hierarchy is fully specified by each cell's position in the crypt column, with position 1 representing the least differentiated cell and position 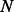 representing the most differentiated cell. In addition, this replacement rule captures the essential features of two biological observations governing cellular repopulation of crypt columns: (i) replacement mitotic activities are concentrated near the stem cell [40], [41]; and (ii) newly divided cells migrate upward in the crypt column [25].

The accumulation of mutations is described by specific transition probabilities between different mutational states. The possibility of back mutations, which reconstitute a less mutated state, is neglected. The mutation rates are assumed to be constant with respect to cell positions and with respect to time. **Table 1** provides the range of plausible values used for the individual mutation rates. In addition to mutations arising during cell division, we also incorporated random mutations not linked to replication into our framework. Due to the lack of data on the positional dependency of this type of random mutation, such mutations are assumed to be equally likely to occur in any position in the crypt column. The mutation rate for the cell division-independent mutation events is considered to be the same as that of mutation events linked to cell division.

**Table 1:** The values used for individual mutation rates.

	u_0_	u_1_	u_2_	u_3_
Type of mutation	*APC* ^+/+^ to *APC* ^+/−^	*APC* ^+/−^ to *APC* ^−/−^	*APC* ^+/+^ to *APC* ^+/+^CIN and *APC* ^+/−^ to *APC* ^+/−^CIN	*APC* ^+/−^CIN to *APC* ^−/−^CIN
Mutation rate per cell division	10^−7^	10^−7^ to 10^−5^	10^−7^ to 2×10^−6^	Up to 10^−2^
References	[49]	[57], [58]	[59]	[15]

Time is measured in rounds of cell shedding. At each elementary time step, the cell at position 80 is shed off into the lumen of the gut as the result of cell division and the associated cell movement. Cell deaths and replenishing cell divisions that replace dead cells are not counted as extra increments in time. The rationale for this design is to distinguish between the regenerative cell divisions under normal circumstances and the compensatory cell divisions for apoptotic losses. The instantaneous time scale for replenishing cell divisions is extrapolated from the observation that under strong external stimuli, the rate of progression through the cell cycle is accelerated; for instance, ionizing radiation was shown to induce proliferative activity [42] and shorten the duration of the cell cycle [43]. In addition, thermal injury [44] and starvation-induced stress [45] also tend to increase mitotic activity. In this study, we adopted the assumption of instantaneous replenishing cell divisions regardless of whether strong external stimuli are present or not.

Relative fitness is defined as the ratio of the proliferation rate of a mutant cell to that of a wild type cell at position *i* in the crypt column, 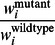. A relative fitness greater than 1 indicates that a mutant cell as position *i* is more likely to be selected to undergo cell division than a wild type cell at the same position. A relative fitness value of less than 1 represents a fitness disadvantage and thus decreases the probability of cell divisions at that position. A relative fitness value of 1 signifies a neutral mutation. We investigated the effects of relative fitness values between 0.5 (representing a 50% fitness disadvantage of a mutant cell) and 2.0 (representing a 100% fitness advantage of a mutant cell) [46].

## Results

### Cellular movement

Using this spatially arranged stochastic process model, we first investigated the effects of different proliferation curves on cell movement. Five different proliferation curves were selected to illustrate their effects on cell movement (**Fig. 1C.**). Curve 1 represents the measured labeling indices (LI) from *in vivo* experiments [23], [42]; curve 2 was selected to investigate the effects of spreading proliferating activities upwards in a crypt column; curve 3 represents the uniform kinetics in which cell proliferation is equally likely to occur in any cell along the crypt column; and curves 4 and 5 represent the mirror images of curves 2 and 1, respectively. The selection probabilities for position 1 in all curves are identical; furthermore the area under each curve is normalized to the same total in order to make the effects of each curve comparable.

**Figure 2:**
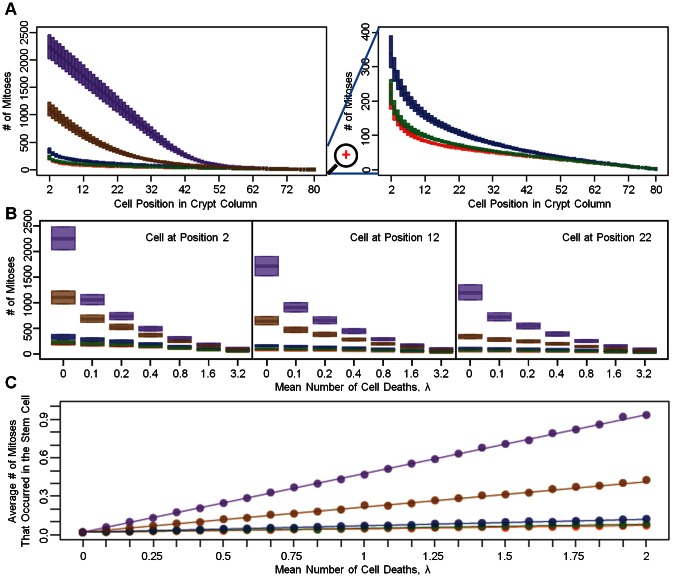
The rate of cellular movement in the crypt column. A: The left panel shows the distribution of the number of mitoses needed to push a cell at position *i* out of the crypt column for each proliferation curve. Colors match the corresponding proliferation curves in Fig. 1C. The horizontal axis represents the position of the cell in the crypt. Box plots indicate the distribution of the number of mitoses, assuming no cell death. The size of each bar indicates the amount of variation in the number of cell divisions. The right panel provides a zoomed-in view focusing on curves 1, 2 and 3. B: The effects of cell death on cellular movement in the crypt for three selected representative positions (2, 12, and 22) in the crypt column under the assumption of a uniform death selection function. As the death rate, *λ*, increases, the rate of cell movement increases, as shown by the decreasing number of mitoses needed to push a cell out of the crypt column. C: The panel shows the effects of cell death on the mitotic stress of the stem cell assuming a uniform death selection function. The average number of times the stem cell is selected for divisions is displayed as a function of cell death for the proliferation kinetic curves. Without cell death, the number of times the stem cell is selected for cell division is identical for all curves. As *λ* increases, the mitotic stress on the stem cell increases. The magnitude of increase depends on the shape of proliferation kinetic curve. Dots represent results from simulations, whereas the lines are exact results based on the terms inside the parentheses in Eq. 8. All graphs are generated based on 1,000 simulations for each kinetic curve under each scenario. All cells are assumed to have identical relative fitness values.

The rates of cellular movement, measured by the number of mitoses required for a cell at a particular position in the column to reach the top of the crypt (position 80), depends on the shape of the proliferation curve (**Fig. 2A** **Left.**). As expected for all kinetic curves investigated, cells located near the bottom of the crypt require a larger number of cell divisions to reach the top. For kinetic curves with mitotic activities concentrated near the base of the crypt column (curves 1 and 2), substantially fewer cell divisions are required to push out a cell located in the bottom half of the column. The numbers of cell divisions required to accomplish this task are very similar for curves 1 and 2. The uniform curve (curve 3) requires slightly more rounds of cell divisions (**Fig. 2A** **Right.**). In contrast, curves 4 and 5, which have mitotic activities concentrated near the top of crypt column, require a much larger number of cell divisions than the other curves. In addition, we observed significant variations among individual runs of the stochastic simulation in the number of cell divisions needed to push a cell out of the crypt when considering the five different curves. At each position in the crypt, curves 4 and 5 show larger variations among simulation runs than curves 1, 2, and 3. Interestingly, cells proliferating according to kinetic curves 4 and 5 show a stronger positional dependency in the amount of variation as compared to curves 1, 2 and 3. For instance, for curves 4 and 5, the number of cell divisions needed to push out a cell located near the top of the crypt column is less variable than that for a cell located near the bottom (**Fig. 2A.**).

In addition to the qualitative descriptions of cellular movement for different growth kinetic curves, we also derived a mathematical representation of the cellular behavior. The dynamics of cellular movement in the crypt column can be represented by a Markov chain. After each round of cell shedding, the transition probability for a cell in position *m* to remain in position *m* is given by 
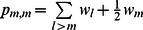
, and transition probability from position *m* to *m*+1 is given by 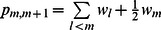 for *m* ranging from 1…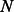, zero elsewhere. The absorbing state for this transition matrix represents the event of a cell exiting the crypt column by reaching its top. The expected number of cell divisions needed for a cell to exit the crypt column (*E*) and the variance of the number of cell divisions (*V*) are given as follows [47]:
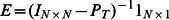
(1)where 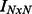 is the identity matrix of size 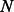, 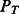 is the transition matrix, and 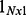 is a vector of length 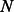 and
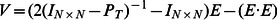
(2)where 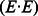 denotes the Hadamard product of the expected number of cell divisions [48]. The concordance between simulation results and results from the Markov chain is shown in **Fig. S1**.

The presence of cell death results in changes in the transition matrix. The new transition matrix is then given by products of the transition matrix for normal shedding, 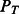 and the transition matrix for replenishing cell divisions, 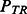. The transition probabilities in the 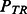 matrix are all zero, except for 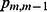, 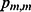 and 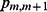, which are given by:
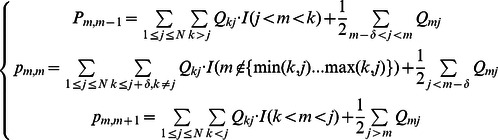
where 
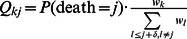
 for *k*≤*j*+δ and *k*≠*j*, zero otherwise; this quantity denotes the probability of apoptosis occurring at position *j* and a replacement cell division occurring at position *k*; and *I* denotes an indicator variable. The overall transition probability matrix is then given by
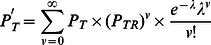
(4)This matrix contains the average transition probabilities in the linear system for a given death rate *λ*. The expected number of cell division needed for a cell to exit the crypt column (*E*) and the variance for the number of cell divisions (*V*) can be calculated from equations 1 and 2. The concordance between the simulation and analytical results calculated using equations 1 and 2 are shown in **Fig. S2**.

The most important effect of cell death is its ability to accelerate cellular movement in the crypt column (**Fig. 2B.**). As the mean number of deaths, *λ*, per cell division increases, fewer rounds of cell division are required to push a cell out of the crypt column (**Fig. 2B.**). These acceleration effects on cellular movement were observed even when *λ* was as low as 0.1 and were more prominent in crypts proliferating according to curves 4 and 5 than those proliferating according to the other curves. Regression analysis indicated that the median number of cell divisions needed to push a cell at position 2 out of the crypt was not significantly different among crypt columns proliferating according to curves 1, 2 and 3. Also, interactions between birth and death curves were not significantly correlated with the speed of cellular movement (see **Table S1**). In addition to causing this acceleration effect, cell deaths also increase the mitotic burden on the stem cell (**Fig. 2C.**). The mitotic burden of a cell is defined as the mean number of times a cell is selected to undergo cell divisions per time step. These divisions could either be cell divisions occurring under normal circumstances or replenishing replications that replace dead cells. Note that, in the absence of cell death (*λ = *0), the average number of times the stem cell is selected to divide is identical for all curves. However, in the presence of cell death, *λ*>0, the number of times of selecting the stem cell to undergo replenishing cell divisions depends on the shapes of the proliferation kinetic and death curves as well as the death rate. Under the uniform death curve, when *λ*>0, proliferative curves 1 and 2 show the smallest increases in the mitotic burden of the stem cell, due to the positioning of the proliferating cells close to the stem cell. These proliferating cells may undergo replenishing cell divisions to replace dead cells arising further up in the crypt column, which help lessen the mitotic burden on the stem cell. In contrast, the stem cell's mitotic burden increases drastically for proliferative curves 4 and 5 due to the long distance between the stem cell and the other proliferating cells. In these cases, cell deaths occurring in the middle of the crypt column can only be replaced by stem cell divisions due to the shape of the mitotic curves and the replenishing rules imposed; this effect thus increases the mitotic burden on the stem cell. The mitotic burden for other cells is less affected by cell death as compared to that of the stem cell and the overall shape of the kinetic curves remains similar to the original shape in the absence of cell death (see **Fig. S3.**). One important consequence of over-using the stem cell to replace dead cells is that it accelerates the rate of mutation accumulation in the stem cell, as explored in the following section.

### The single mutation model

**Figure 3:**
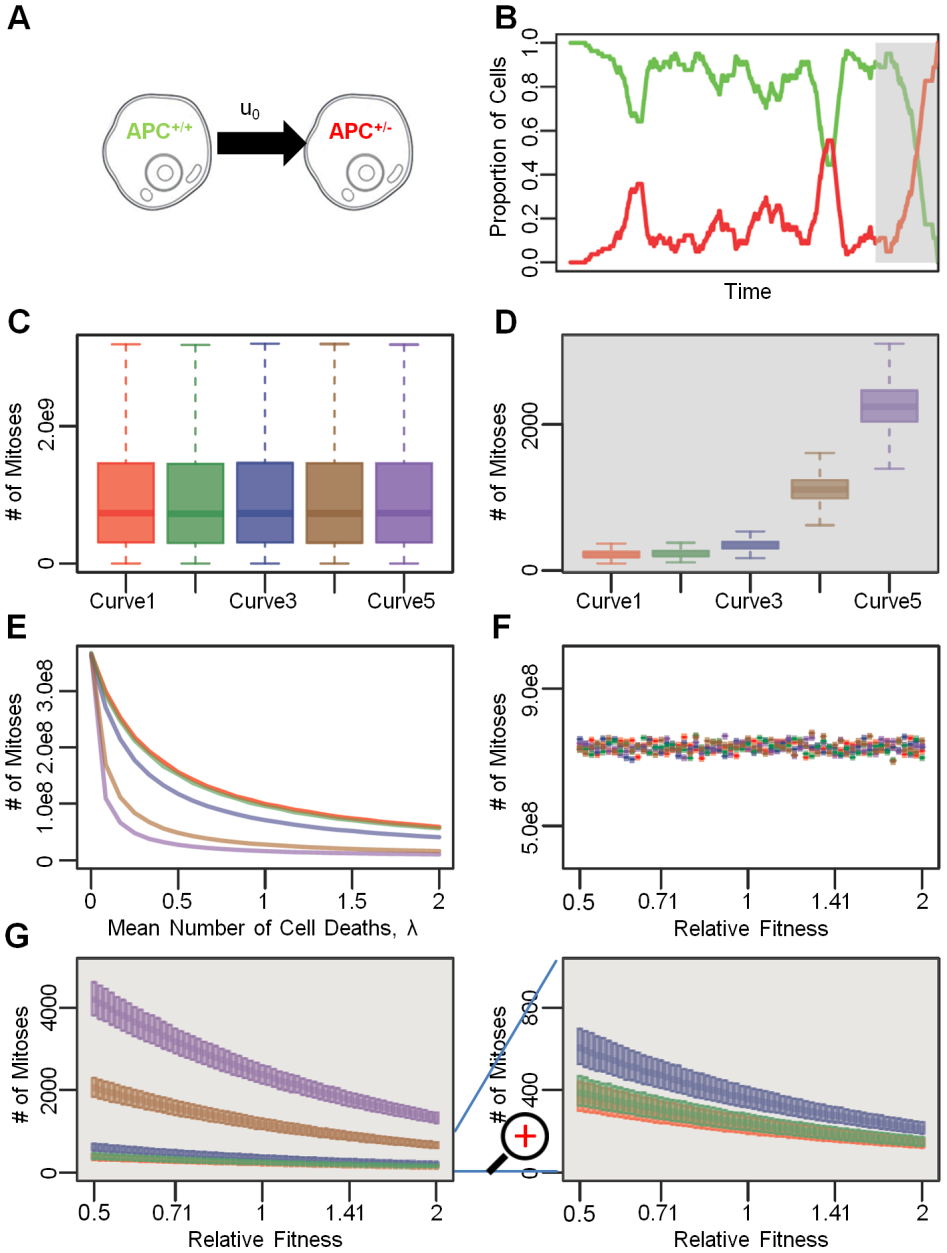
The single mutation model. A: Schematic representation of the single mutation model. B: An example of the dynamics of somatic evolution in the crypt column. The curves show the proportions of cells in the crypt column. The colors correspond to cell types in panel A. The gray shaded region indicates the stem cell is mutated to *APC* ^+/−^. For illustration purposes, u_0_ = 0.01. C: The number of mitoses needed for a wild type crypt column to transition to an *APC* ^+/−^ state for various proliferation kinetic curves in the absence of cell death. As expected, the numbers of cell division needed to reach mutant fixation are the same for all curves. The mutation rate is *u*_0_ = 10^−7^ per cell division. Box plots are color coded, corresponding to the curves in Fig. 1C. D: The number of mitoses needed for fixation of *APC* ^+/−^ cells for various proliferation kinetic curves, measured from the time at which the stem cell accumulates the *APC* ^+/−^ mutation assuming no cell death. The gray area corresponds to the gray shaded interval in panel B. E: Acceleration of mutation accumulation due to cell death. As the death rate, *λ*, increases, fewer cell divisions are required for a mutated stem cell to arise. The comparison between panels C–E highlights the importance of proliferation kinetics of non-stem cells in the presence of cell death. F: Effects of fitness differences and proliferation curves on the rate of somatic evolution. The range of relative fitness spans from 0.5 to 2.0. G: The left panel shows the effects of fitness differences and different proliferation curves on the rate of *APC* ^+/−^ fixation, starting from an *APC* ^+/−^ stem cell in the absence of cell death. The panel on the right provides a zoomed-in view on curves 1, 2 and 3.

We then incorporated a simple mutation model into the linear process. This addition captures for instance the accumulation of an inactivating mutation in one *APC* allele, thus transforming an *APC* ^+/+^ cell into an *APC* ^+/−^ cell (**Fig. 3A.**). As a representative example, the dynamics of the somatic evolution process within a single crypt column is displayed in **Fig. 3B**. In the absence of cell death, all mutations arising in non-stem cells within the crypt column are eventually flushed out of the crypt; only mutations arising in the stem cell have the ability to reach fixation, i.e. reach 100% frequency within a crypt column. This spatial restriction highlights the fundamental difference between the linear process and a stochastic process model describing a well-mixed population, for instance the Moran process [28] or the Wright-Fisher process. In the latter models, any cell within the population has an equal chance of taking over the population if all fitness values (i.e. growth and death rates) are equal. In the linear process, in contrast, only a mutation arising in the stem cell has the ability to take over the entire population of cells.

Somatic evolution in the crypt column in the absence of cell death can be split into two disjoint events: (i) a mutation arising in the stem cell, and (ii) mutation propagation through the crypt column (**Fig. 3B.**). Since the mutation rate at which *APC* is inactivated per allele, *u_0_*, is low (estimated to be on the order of 10^−7^ per cell division [49]), the rate-limiting event of this process is the time it takes until the mutation arises in the stem cell; mutations arising in any other cell are neglected here since they cannot reach fixation in the crypt. The number of times the stem cell divides is specified by the proliferation kinetic curve. In the absence of cell death, the probability of selecting the stem cell for cell division is identical for all kinetic curves; thus the time, measured by the number of mitoses occurring, until all cells in the crypt column are in the *APC* ^+/−^ state is identical for all kinetic curves (**Fig. 3C.**). The distribution of mitotic activities along the crypt column plays no role in modifying the speed of mutation accumulation in the stem cell, since the rate of stem cell divisions are equal among all curves investigated (see **Methods**). The probability of fixation by time *t* in the linear process is determined by the probability that a mutation has arisen in the stem cell by time *t*,
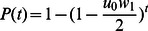
(5)For a small *u_0_*, this expression can be approximated by
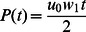
(6)Here 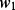 denotes the probability of selecting the stem cell position for replication and the factor of ½ arises from the two possible arrangements of cells after cell divisions, only one of which can lead to a mutant cell residing in the stem cell position. Once the stem cell becomes mutated, fixation of its offspring in the crypt column quickly ensues. However, the rate of propagation of mutated cells throughout the crypt is heavily influenced by the kinetic curves (**Fig. 3D.**). Mutated cells in crypts proliferating according to curves 1 and 2 reach fixation faster than in those proliferating according to curves 4 and 5. Curve 3, the uniform curve, leads to fixation of mutant cells on a slightly slower time scale than curves 1 and 2, but still faster than curves 4 and 5. In addition, the amount of variability in the number of cell divisions required for the *APC* ^+/−^ mutation to propagate through the crypt column also depends on the shapes of the kinetic curves. As the mitotic activity becomes more concentrated near the top of the crypt, towards the gut lumen, the amount of variability among individual simulation runs in the fixation time increases (**Fig. 3D.**).

In contrast, in the presence of non-stem cell death, the interactions between birth and death curves determine the speed of somatic evolution (**Fig. 3E.**). We derived an analytical approximation to show the accelerating effects of non-stem cell death on the rate of mutation accumulation in the stem cell, in the absence of relative fitness differences between mutated and normal cells and in the absence of stem cell death. The probability of selecting the stem cell to undergo one round of additional cell division, and thus to shift all downstream cells along the crypt column to fill the vacancy created by a cell death at position *j*, is given by 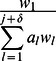, where *δ* specifies the size of the interval (i.e. number of cells) in which more differentiated cells can replace the dead cell. Thus, the probability of a mutation arising in the stem cell during this additional round of cell division is given by 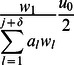. Since the expected number of cell deaths occurring during a time interval *t* is 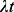, the probability that a mutation occurs in the stem cell during 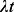 additional cell divisions is given by
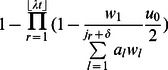
(7)where 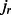 indicates the position at which the *r* ^th^ cell death event occurs. For small 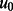, this expression can be approximated by 
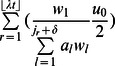
. If 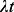
>>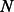, it can further be approximated by 
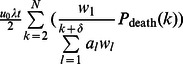
, where 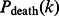 denotes the probability of cell death occurring at a particular position in the crypt column, and 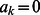.

In addition to division-linked mutations, cell division-independent mutation also contributes to the rate of mutation accumulation in the stem cell. Given the assumption that cell division-independent mutation may occur anywhere in the crypt column, the additional contribution to the overall rate of mutation accumulation in this scenario can be modeled by including the factor 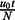. Thus the overall fixation rate in the crypt column of a new mutant cell is given by
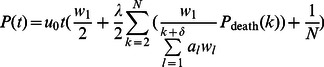
(8)


The interactions between birth kinetics and death positions determine the probability of selecting the stem cell for replenishing cell divisions after apoptosis, as stated by the term 
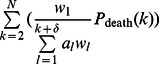
 in equation (8). A comparison between the analytical approximation using equation (8) and simulations is shown in **Fig. S4**.

For the five curves examined, the interactions between birth kinetics and death selection are shown in **Table 2**. Curve 1 has the lowest probability of selecting the stem cell for replenishing cell divisions for all death curves. In addition, the rates of selecting the stem cell are less variable for curve 1 across different death selection curves (row 1 in **Table 2**) than for other proliferation kinetic curves. Deaths occurring near the top of the crypt column, such as in death curve 5, lower the mitotic burden on the stem cell for all birth kinetics curves. Of all twenty-five combinations examined, the interaction between birth kinetics curve 1 and death selection curve 5 results in the lowest amount of mitotic burden on the stem cell. In contrast, birth kinetics curve 5, with mitotic probabilities concentrated near the top of the crypt, and death selection curve 1, with apoptosis occurring near the stem cell, result in substantial increases in the probability of selecting the stem cell for replenishing cell divisions. In addition to the interaction between the location of proliferating cells and cells undergoing apoptosis, the rate of cell death, λ, also controls the rate of mutation accumulation in the stem cell. As λ increases, the rate of mutation accumulation in the stem cell is accelerated. Lastly, cell division-independent mutation increases the overall mutation rate, as shown by the last term in equation (8).

**Table 2:** The probabilities of selecting the stem cell for a replenishing division per cell death event.

	Death Curve 1	Death Curve 2	Death Curve 3	Death Curve 4	Death Curve 5
Birth Curve 1	0.0374	0.0344	0.0269	0.0193	0.0186
Birth Curve 2	0.0471	0.0421	0.0314	0.0207	0.0190
Birth Curve 3	0.0844	0.0735	0.0504	0.0274	0.0224
Birth Curve 4	0.4201	0.3354	0.1969	0.0584	0.0293
Birth Curve 5	0.9180	0.7525	0.4587	0.1650	0.0488

The data is shown for all combinations of birth and death kinetics in the absence of stem cell death and of fitness differences among mutant cells. The five birth curves are the measured curve from the labeling index, the logistic curve, the uniform curve, and, mirror images of the measured curve and logistics curve with the place of reflection between cell 40 and 41, respectively. Due to the lack of quantitative measurements of the distribution of death as a function of cell position, birth curves are used as proxies for death curves.

We also investigated the effects of fitness changes of mutant cells on the speed of somatic evolution. Relative fitness is defined as the ratio of selection probabilities for proliferation of a mutant cell to that of a wild type cell at the same position in the crypt column (see **Methods**). Changes in the relative fitness can potentially affect the speed of both the rate of mutation accumulation in the stem cell and the rate of mutant propagation through the crypt column. We investigated the effects of relative fitness values between 0.5 (representing a 50% fitness disadvantage of a mutant cell) and 2.0 (representing a 100% fitness advantage of a mutant cell) [46]. The speed of somatic evolution was not significantly affected by changes in fitness within this range (**Fig. 3F.**). This effect arises because the rate-limiting event is represented by the generation of the first mutation in the stem cell, which – unlike its propagation throughout the crypt column – is not dependent on fitness. In the case of an extremely advantageous mutation with a relative fitness value greater than 10, fixation of its offspring in the crypt column can be substantially delayed for the cases in which the initial mutation arises in a non-stem cell. In these cases, cell divisions in non-stem mutants are driving the cellular movement and tissue regeneration in the crypt column, thus reducing the probability of the stem cell undergoing cell divisions. When conditioning on the event that the stem cell is already mutated, we found that the relative fitness has a significant effect on the fixation time for some kinetic curves (**Fig. 3G.**). For instance, curves 4 and 5 are more sensitive to changes in relative fitness; as the relative fitness of mutants decreases, more cell divisions are required for a mutant cell to reach fixation. In contrast, curves 1 and 2 are less sensitive to changes in the relative fitness. The variation in the fixation time also depends on the kinetic curves and on the relative fitness of the mutant cells. Again, curves 1 and 2 lead to less variation compared to curves 4 and 5. Furthermore, at low relative fitness values, the number of cell divisions necessary for fixation is more variable than at high relative fitness values for curves 4 and 5. The uniform curve is more sensitive to fitness variations compared to curves 1 and 2 (**Fig. 3G.**).

We then modified the basic mathematical framework to allow for the special case of stem cell death. In such situations, a mutant stem cell may be replaced by a more differentiated cell. Apoptosis of the stem cell delays the rate of mutation accumulation in the stem cell and mutant fixation. Regression analysis indicates that the number of times a mutant stem cell is replaced by a wild type cell as the result of apoptosis in the stem cell position is significantly related to the birth kinetics, death rate, relative fitness, and specific interactions between birth and death curves (see **Table S2**). Kinetics curves with mitotic probabilities concentrated near the top of the crypt (curves 3, 4 and 5) are less likely to lead to a loss of a mutation that had arisen in the stem cell position. Mutant stem cells with a low relative fitness are more likely to be replaced by wild type cells, since such mutant cells have a slower rate of propagation. Furthermore, large death rates increase the mean number of times a mutant stem cell is replaced by a wild type cell through apoptosis and dedifferentiation. Since the death selection function is normalized such that the stem position has an equal probability of being selected for all curves, death curves have no effect on the rate at which mutant stem cells are replaced by wild type cells. The interactions between birth and death curves only have weak effects on how often the mutant stem cell loses a mutation. Overall, these observations suggest that apoptosis in the stem cell has the ability to delay mutant fixation, whereas apoptosis in the differentiated cells accelerates mutation accumulation by increasing stem cell divisions. Consistent with this observation, recent studies suggest that a high rate of stem cell apoptosis in the small intestine is partially responsible for the low incidence of small intestine cancer compared to colorectal cancer; in the latter, the stem cell is protected from undergoing apoptosis by the expression of *bcl-2*, an anti-apoptosis protein [50] .

### The two mutation model

**Figure 4:**
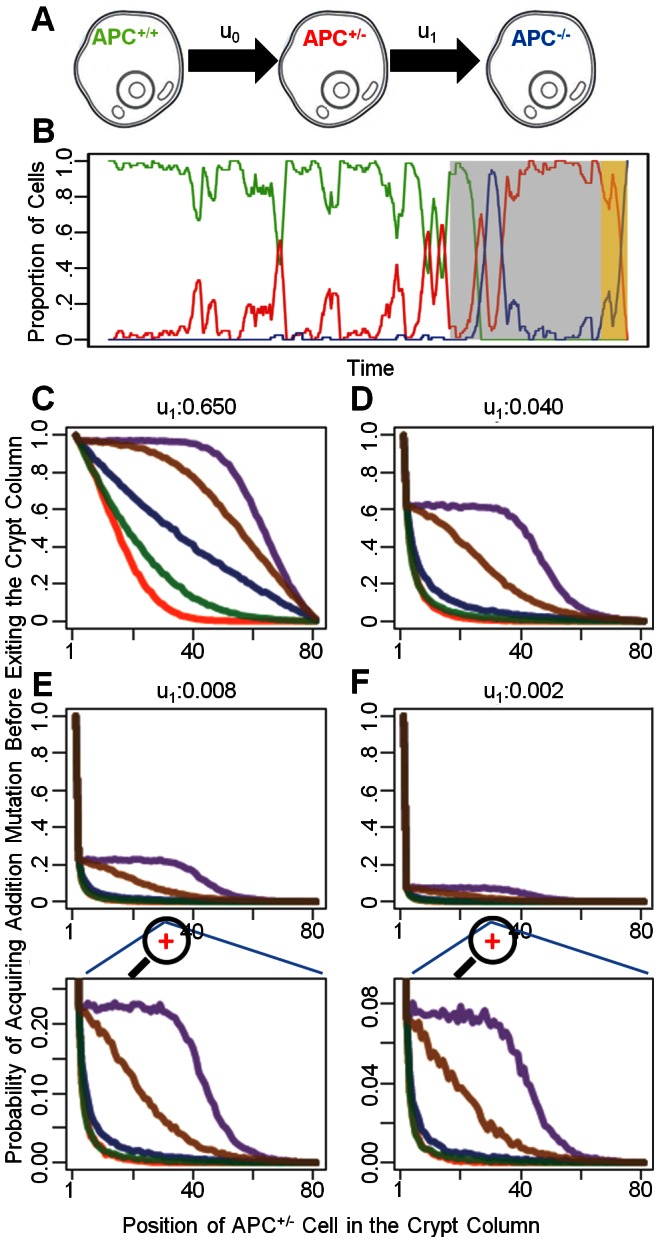
The two mutation model. A: Schematic representation of the mutations leading to inactivation of both *APC* alleles, at rates *u*_0_ and *u_1_*, respectively. B: A representative example of the dynamics of somatic evolution in the linear process. Each color-coded curve represents the proportion of each cell type in the crypt column, with colors corresponding to those in panel A. The gray shaded region represents the time interval during which the stem cell is in the *APC* ^+/−^ state. The yellow shaded region represents the time interval during which the stem cell is in the *APC* ^−/−^ state. C–F: Conditional probability for losing the second *APC* allele before an *APC* ^+/−^ cell at a particular position along the crypt column is “flushed” out of the crypt in the absence of cell death. At each position between 2 and 80, 1,000 simulation runs are generated in the absence of cell death to determine the probabilities of *APC* ^+/−^ cells gaining new mutations before being “flushed” out of the crypt column. Four different rates of inactivating the second *APC* allele, *u*_1_, were investigated and are shown on the top of the four sub panels. The bottom two panels provide zoomed-in views.

We then investigated a two mutation model within the linear process. This scenario captures, for instance, the mutations inactivating both alleles of the *APC* gene (**Fig. 4A.**). Similarly to the single mutation model, the rate of emergence of double mutant cells (i.e. *APC* ^−/−^ cells) is driven by the rates per cell division at which the two mutations arise, for all growth kinetic curves investigated.

We first studied the dynamics of the accumulation of these two mutations by investigating the effects of proliferation kinetics on the probability that an *APC* ^+/−^ cell acquires an additional mutation in the second *APC* allele before being flushed out of the crypt. We investigated these dynamics in the absence of cell death. **Fig. 4B** **.** shows a representative simulation run. There are two scenarios: the first *APC* mutation may arise in the stem cell, or alternatively it could arise in a non-stem cell. Once the stem cell harbors the first *APC* mutation, in the absence of cell death, this mutation is permanently maintained in the crypt and eventually, the second *APC* mutation arises. In contrast, if the first *APC* mutation arises in a non-stem cell, the probability that this cell and its progeny gain an additional mutation before exiting the crypt depends on four factors: the mutation rate for inactivating the second *APC* allele, *u_1_*, the location of the *APC* ^+/−^ cell, the kinetic curve, and the relative fitness of the *APC* ^+/−^ cell. As expected, an increase in *u_1_* enhances the probability of accumulating an additional mutation in a non-stem mutant cell (**Fig. 4C–F.**). This observation agrees with the findings by Komarova and Wang [27] that for a large *u_1_*, *APC* ^+/−^ cells are likely to arise among differentiated cells. The position of the *APC* ^+/−^ cell also has a significant influence on the probability of accumulating the additional mutation. An *APC* ^+/−^ cell residing near the stem cell has a higher probability of acquiring a second mutation before being “washed” out of a crypt than a cell residing near the top of crypt (**Fig. 4C–F.**). Finally, different birth kinetic curves also have effects on the probability of acquiring further mutations. For an *APC* ^+/−^ cell at a particular position between 3 and 80, curves with mitotic activities concentrated near the stem cell (curves 1, 2 and 3) have small probabilities of acquiring the second *APC* mutation before the cell exits the lattice (**Fig. 4C–F.**). This effect is more prominent for a large than for a small *u_1_*. The probability of accumulating the second mutation depends on the sum of mitotic probability weights for each cell: *w_1_* +…+*w_i-1_*, where *i* denotes the position of the first *APC* ^+/−^ cell; this sum is inversely related to the probability of accumulating the second *APC* mutation. For instance, if the *APC* ^+/−^ cell resides at position 8, then the curve with the smallest sum of mitotic probability weights for positions 1 to 7 leads to the largest chance that this clone accumulates an additional mutation before exiting the lattice. Since all curves have the same mitotic probability for position 1, the chance that an *APC* ^+/−^ cell at position 2 accumulates a new mutation is the same for all kinetic curves. This effect arises only in the absence of cell death. Therefore, conditional to the event that an *APC* ^+/−^ cell resides between positions 3 and 80, those kinetic curves with mitotic probability weights concentrated near the stem cell confer a protective effect against acquiring new mutations. Increasing the relative fitness of mutant cells increases the likelihood that an *APC* ^+/−^ cell acquires a new mutation by enhancing the likelihood of this cell and its progeny to be selected for cell divisions. These considerations hold under the assumption of no cell death. The presence of cell death, in contrast, reduces the probability for a mutant cell to gain an additional mutation; increasing the rate of cell death enhances the likelihood for a mutant cell and its progeny to either die or be flushed out the crypt before they accumulate a new mutation.

### Chromosomal instability

**Figure 5:**
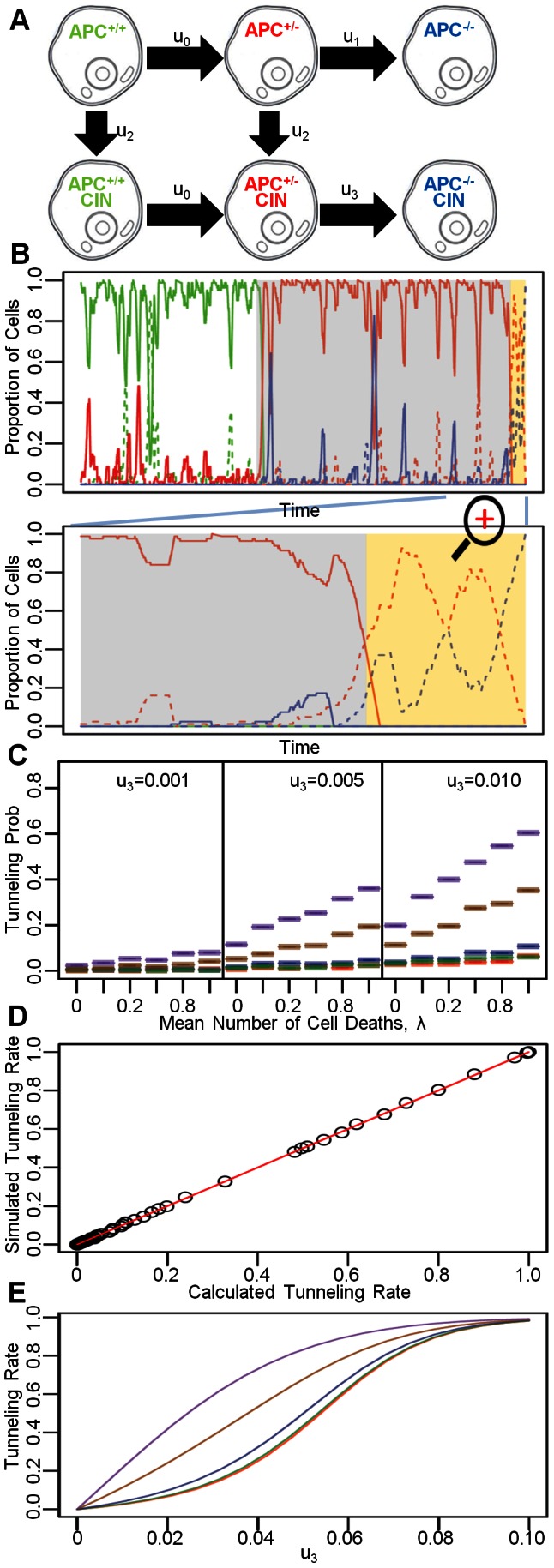
The effects of chromosomal instability and tunneling. A: Schematic representation of the mutations leading to the inactivation of both *APC* alleles incorporating chromosomal instability. B: The upper panel displays a representative example of linear somatic evolution dynamics. Each color-coded curves represent the proportion of each cell type in the crypt column, with colors corresponding to those in panel A. Dashed and solid lines correspond to cells with and within CIN respectively. The gray and yellow shaded regions represent the time interval during which the stem cell is in the *APC* ^+/−^ state and *APC* ^−/−^ state respectively, regardless of CIN status. The lower panel provides a zoomed-in view. Notice that the dashed red curve does not reach 1, which signifies tunneling. This representative simulation run is performed using the uniform proliferation kinetics in the absence of cell death and fitness differences. The mutation rate is inflated to *u*_0_ = *u_1_* = *u_2_* = 10^−3^ and *u_3_* = 0.01 for computational speed. C: The tunneling probability as a function of *u_3_* and the cell death rate, *λ*, for the five proliferation curves under uniform death selection. To reduce the extent of complexity, tunneling rate is simulated using a three-state system consisting of *APC* ^+/−^, *APC* ^+/−^ CIN and *APC* ^−/−^ CIN cells instead of the six-state system as illustrated in Panel A. Each simulation run starts with a crypt column seeded with an *APC* ^+/−^ cell at the stem position. The number of simulation runs is set at 1,000. Stem cell death is allowed. D: Concordance between simulated tunneling rates and analytical rates for linear systems of length *N* = 10, 20, …100 with equal proliferation probability at each position, using mutation rate *u*_3_ = 0.001, 0.01, 0.1 and 1.0. All simulations were performed for 1,000 runs. E: Analytical tunneling rate for the five proliferation curves at different mutation rate.

Finally, we incorporated the effects of chromosomal instability (CIN) into our model. CIN arises due to the accumulation of a specific mutation at rate *u_2_* and leads to a large mutation rate, *u_3_*, at which the second *APC* allele is inactivated during cell divisions [15] (**Fig. 5A.**). Considering this additional mutation event, we then set out to investigate the effects of CIN on the dynamics of the system. For large *u_3_*, we observed a phenomenon that has previously been termed “stochastic tunneling” [51] . Tunneling refers to the process in which a crypt column moves from a homogeneous state in which all cells harbor *ρ* mutations to a homogeneous state in which all cells harbor *ρ*+2 mutations, without ever transiting through a state in which all cells harbor *ρ*+1 mutations. For instance, in our model, tunneling occurs when cells in the crypt column move from an *APC* ^+/−^ state directly to an *APC* ^−/−^CIN state, without reaching fixation in the *APC* ^+/−^ CIN state, as illustrated in **Fig. 5B**. Another possible tunneling scenario is *APC* ^+/+^ CIN to *APC* ^+/−^ CIN to *APC* ^−/−^ CIN. Tunneling between other states is less likely to occur given the small second mutation rates. Unlike prior investigations concerning the tunneling rate for a well-mixed population of cells [52] , cells in this model remain constrained to a one-dimensional lattice.

The tunneling rate depends on the mutation rate, *u_3_*, the death rate, the birth kinetic curve, the death selection curve, and the relative fitness of mutated cells (see **Table S3**). Consider the uniform death curve (curve 3) as an illustration: for this curve, as *u_3_* increases, a larger proportion of all simulation runs display tunneling for all five proliferation kinetic curves (**Fig. 5C.**). Even though all curves lead to this phenomenon, we found that crypts proliferating according to curves 4 and 5 are more likely to display tunneling than those proliferating according to curves 1, 2 and 3. For curves 4 and 5, more stem cell divisions are required to reach fixation of *APC* ^+/−^ CIN cells, starting from an *APC* ^+/−^ CIN stem cell, than for the other curves. Because of the large number of stem cell divisions in crypts proliferating according to curves 4 and 5, there is a large probability for the stem cell to lose the second copy of the *APC* gene before all cells in the column become *APC* ^+/−^ CIN cells. For a given *u*_3_, as the mean number of cell deaths, *λ*, increases, a larger proportion of simulation runs reach fixation of *APC* ^−/−^CIN cells via tunneling. Furthermore, we found that increases in *λ* result in larger increases in the tunneling probability for cells proliferating according to curves 4 and 5 than for those proliferating according to curves 1, 2 and 3. Finally, in the presence of cell death, high relative fitness values of *APC* ^+/−^ CIN cells reduce the tunneling probability, whereas high relative fitness values of *APC* ^−/−^ CIN cells promote tunneling. Since the relative fitness values of *APC* ^+/−^ CIN and *APC* ^−/−^ CIN cells are correlated, the ratio of relative fitness values of *APC* ^+/−^ CIN and *APC* ^−/−^ CIN cells determines the tunneling rate. This ratio affects the selection of replacing cell divisions and hence the tunneling probability. Higher-order interaction terms involving birth curves, death curves and death rate are also important determinants of tunneling rates (see **Table S3**).

Finally, we derived a general solution for the tunneling probability under the assumption of no cell death. The general solution comprises the product of three terms: (1) the limiting probability of the initial *APC* ^−/−^ CIN cell at position *k* to reach either of the absorbing states: that an *APC* ^−/−^ CIN stem cell arises, leading to tunneling, or that no further mutation arises in the stem cell position before the *APC* ^−/−^ CIN clone is removed from the crypt column, signifying the fixation of *APC* ^+/−^ CIN cells; (2) the probability that the initial *APC* ^−/−^ CIN cell arises at position *k* conditional to the event that an *APC* ^+/−^ CIN to *APC* ^−/−^ CIN mutation occurred while *APC* ^+/−^ CIN cells populating the crypt column reach position *l*; (3) the probability that an *APC* ^+/−^ CIN to *APC* ^−/−^ CIN mutation occurs as *APC* ^+/−^ CIN cells populating the crypt column reach position *l*. The transition matrix for calculating the limiting probabilities is given by
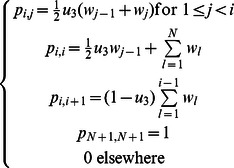
(9)


The transition matrix specifies the movement of the left-most *APC* ^−/−^ CIN cell in the crypt column. Under the assumption of no cell death, the movement of the left-most *APC* ^−/−^ CIN cell completely determines the tunneling probabilities. The two absorbing states are state 1, when the left-most *APC* ^−/−^ CIN cell arises in the stem cell position, resulting in tunneling, and state 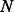
+1, when the left-most *APC* ^−/−^ CIN cell is out of the crypt column, resulting in fixation of *APC* ^+/−^ CIN cells. The tunneling probability vector of length 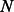
−1 can be calculated using the fundamental matrix of an absorbing Markov chain [47], [48],
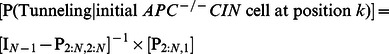
(10)Thus, the overall tunneling vector of length 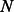, denoted by [P(T|*k*)], is given by
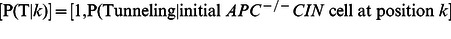
(11)This expression arises since tunneling occurs with probability 1 if the *APC* ^+/−^ CIN to *APC* ^−/−^ CIN mutation arises in the stem cell.

Under the assumption of no cell death, the mutation conferring CIN must occur in the stem cell position, since otherwise, it would be flushed out of the crypt column. Thus, the probability for the initial *APC* ^−/−^CIN cell to arise at position *k* depends on the position of the right-most *APC* ^+/−^CIN cell as the latter populate the crypt column:
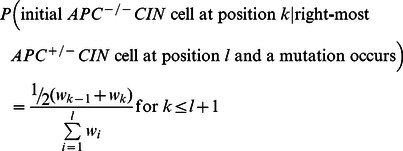
(12)Thus, the probability that the initial *APC* ^−/−^CIN cell occurs at position *k* conditional to the event that the right-most *APC* ^+/−^CIN cell is located at position *l* and a mutation occurs can be arranged in matrix form, denoted by [P(*k*|*l*)], with columns representing *k* and rows representing *l*. Lastly, the probability that an *APC* ^+/−^ CIN to *APC* ^−/−^ CIN mutation occurs as the right-most *APC* ^+/−^ *CI* 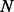 cell moves into position *l* follows a geometric distribution:
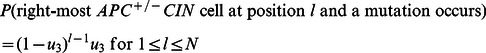
(13)Thus, a vector of length 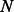, denoted by [P(*l*)], completely specifies the probabilities of a mutation occurring as the right-most *APC* ^+/−^CIN cell moves from *l*-1 position to *l.* Therefore, the overall tunneling probability can be calculated as
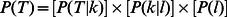
(14)


We then compared this analytical result with exact computer simulations and found good agreement (**Fig. 5D.**). Furthermore, using this analytical result, we calculated the tunneling rates for the different proliferation curves at various values of the *APC* ^+/−^ CIN to *APC* ^−/−^ CIN mutation rate *u_3_* (**Fig. 5E.**). For a given *u_3_*, those proliferation curves with mitotic probabilities concentrated near the stem cell have lower tunneling probabilities than those with mitotic probabilities near the top of the crypt. In addition, in the absence of cell death, a variation in fitness values does not affect the tunneling rates (**Fig. S5.**); in contrast, fitness variation is significantly associated with the tunneling rate when cell death is present (**Table S3**).

## Discussion

We designed a spatially arranged computational model of intestinal epithelial cells to investigate the effects of proliferation kinetics on the dynamics of cell movement and mutation accumulation in the colonic crypt. The model considers a single cell column within a colonic crypt, in which 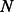 cells are arranged onto a one-dimensional lattice. One end of the lattice represents the bottom of the crypt, where the stem cell resides, and the other end represents the orifice of the crypt (**Fig. 1A.**). Mitotic activities cause cell movements towards the upper end of the crypt and push the last cell off the lattice. During each cell division, a mutation may occur, which might increase the proliferative activity of the resulting mutant cell and can represent a step towards colorectal tumorigenesis. We then compared the effects of five different proliferation curves on the speed of somatic evolution and cell movement (**Fig. 1C.**). We used these proliferative curves to demonstrate that proliferation kinetics is an important criterion that needs to be considered in modeling the dynamics of somatic evolution in spatially arranged tissues. In addition to the proliferation kinetics, we introduced a differentiation hierarchy to this linear process model. In our model, only cells at similar or less differentiated stages can replace dead cells. Finally, we used a discrete time scale such that under normal circumstances, only cell divisions contribute to the measurement of time while cell death and replenishing cell division events are assumed to be instantaneous in time.

Compared to previously published models [33], [34], [36], [37], in our model the crypt structure is greatly simplified to a one-dimensional lattice; nonetheless, the essential features of cell movement are captured by our simple model, in that cells move upward towards the gut lumen with limited lateral movement [22]. This simplified design allows for the investigation of the effects of proliferation kinetics on the rate of mutation accumulation. In addition, unlike previously published models, our simple design enables us to derive analytical solutions for several quantities of interest such as the rate of mutation accumulation and the tunneling probability. In contrast to compartmental models [53]–[55] , the linear model has the advantage of retaining the spatial structure dictating colonic epithelial cell behavior. In addition, our model contains a gradual differentiation hierarchy, which is represented by the cell positions in the crypt, instead of being characterized by discrete compartmental boundaries.

Using this model, we demonstrated that spatially explicit proliferation kinetics have a significant impact on the stability and the dynamics of the crypt column in terms of the speed of cell movement and mutation propagation as well as sensitivities to apoptosis and selective effects of mutant cells. Comparing the proliferation kinetic curves we investigated, we identified three advantages of a spatial architecture in which the proliferative potential of cells is located close to the stem cell: 1) this type of proliferation architecture increases the stability of the linear system in terms of providing a less variable rate of cell movement; 2) in the presence of cell death, this architecture delays the rate of mutation accumulation in the stem cell; and 3) it provides protection against tumorigenesis by reducing the probability of acquiring further mutations in the absence of cell death. These results suggest that the kinetic curve identified using labeling index studies in the human colon [19], [23] best delays the rate of somatic evolution towards colorectal tumorigenesis when compared to the other curves investigated here.

Prior work has demonstrated that both spatial organization and cellular hierarchy need to be considered in modeling somatic evolution [56] . Our findings highlight the importance of proliferation patterns, in addition to spatial arrangements and cellular hierarchies, in studying tissue and mutation dynamics. This area has not been explored in-depth prior to our investigation. Despite the highly simplified nature of our model, we have demonstrated that proliferation curves with mitotic activities concentrated near the stem cell confer an advantage to the colon crypt by increasing the stability of the linear system and by delaying the rate of mutation accumulation. Normal proliferation kinetics, in addition to the linear tissue architecture, can suppress the rate of evolution towards colorectal cancer. A departure from such proliferation kinetics accelerates the rate of mutation accumulation in the colonic crypt and destabilizes natural cell flow, thus representing a step towards cancer.

## Supporting Information

Figure S1 **Concordance between results and analytical results from Markov chain in the absence of cell death.** Top row: the average number of cell divisions needed for cells at positions 2–80 to exit the crypt column. Each dot represents a position (2–80) in crypt column. Bottom row: the variance associated with the number of cell divisions. Simulated results are based on 1,000 simulations. As expected simulated results and analytical results fall on a straight line. Color corresponds to the different proliferation curves in Fig. 1.
(TIF)

Figure S2 **Concordance between simulated and analytical results from Markov chain in the presence of cell death.** A: the average number of cell divisions needed for cells at positions 2–80 to exit the crypt column. Each row represents a particular death rate, λ, from top to bottom, λ = 0.1, 0.2, 0.4, 0.8, 1.6 and 3.2. B: the variance associated with the number of cell divisions for λ = 0.1, 0.2, 0.4, 0.8, 1.6 and 3.2. As expected simulated results and analytical results fall on a straight line. Color corresponds to the different proliferation curves in Fig. 1. Simulated results are based on 1,000 simulations.
(TIF)

Figure S3 **Overall proliferation curves for various combinations of proliferation, death curves and death rate.** Each row represents a proliferation curves, curve 1 on the top and curve 5 at the bottom. Each column represents a particular death selection curve, death curve 1 on the left and death curve 5 on the right. Seven death rates (λ) are tested ranging from 0 to 3.2. Cell death and replenishing cell divisions results in deviations from the original proliferation curve (λ = 0).
(TIF)

Figure S4 **Concordance between simulated and analytical rates of somatic evolution.** Comparison between the simulated somatic evolution rate and analytical approximated rate (Eq. 8), using *u*_0_ = 0.01, proliferation kinetics curve 5, death curve 3 and death rate *λ* = 0.5 with 1,000 simulation runs. The red line indicates the analytically approximated results and black dots indicate the simulated results. The number of cell divisions is truncated at time 600 to compensate for the artificially high mutation rate (*u*_0_ = 0.01) used to speed up the simulation. The cut of time at 600 is selected based on the number of cell divisions in a crypt column: 24 division/day × 365 days/year × 70 years =  613,200 divisions per lifetime; and to compensate for difference between *u*_0_ used in the simulation and the actual value, *u*_0_ = 10^−7^, such that we have 613,200/(0.01/10^−7^) = 6.13. This number of divisions is scaled up by a factor of 100 to demonstrate the robustness of Eq. 8.
(TIF)

Figure S5
**Tunneling probabilities are not sensitive to fitness variation in the absence of cell death.** X-axis shows the calculated tunneling probabilities using Eq (14) which does not require relative fitness values as inputs. Y-axis shows the simulated tunneling probabilities. Simulated tunneling probabilities are simulated using linear systems of length 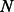 = 10, … 100 with equal proliferation probability at each position, mutation rate *u*_3_ = 0.001 …1.0 and relative fitness values of 1.0, 1.5 and 1.0 for APC^+/−^, APC^+/−^CIN and APC^−/−^CIN cells respectively. Simulated results are based on 1,000 simulations.(TIF)

Table S1 **Parameters associated with cell movement.** Regression coefficients associated with the median speed of cell movement for a cell at position 2 in 1,000 simulations for each combination of the 5 proliferation kinetic curves, 5 death selection curves, and 4 death rates (0, 0.01, 0.1, and 2). Significantly associated covariates are highlighted (p-value<0.05). Positive estimates indicate that more cell divisions are needed for the cell at position 2 to exit the crypt column. Negative estimates indicate that fewer cell divisions are needed. The associations between the median speed of cell movement and the parameters are assessed using linear regression. The linear regression (least square fitting) was performed with the median number of mitoses needed to exit the column for a cell located at position 2 as the dependent variable, and the kinetics curves (categorical variables), death selection curves (categorical variables), the interaction between proliferation and death curves (categorical variables) and death rate as independent variables. Categorical variables are incorporated into the regression model by indicator design matrix with curve 1 as baseline. The interaction terms are delimited by semicolons.
(XLSX)

Table S2 **Parameters associated with the probability of death of a mutated stem cell.** Regression coefficients associated with the number of times a mutant stem cell is replaced by a wild type cell through stem cell apoptosis in 1,000 simulations for each combination of the 5 proliferation kinetic curves, 5 death selection curves, 4 death rates (0, 0.01, 0.1, 2), and 3 fitness values (0.5, 1.0, 2.0). Significantly associated covariates are highlighted (p-value<0.05). Positive estimates indicate that more loss of mutant stem cells is associated with the parameter. Negative estimates indicate less mutated stem cell loss is associated with the parameter. The associations between the number of times a mutated stem cell dies and the effects of input parameters are assessed using linear regression. The linear regression (least square fitting) was performed with the number of times a mutated stem cell dies as the dependent variable, and proliferation kinetic curves (categorical variables), death selection curves (categorical variables), the interaction between proliferation and death curves (categorical variables), death rate, and relative fitness of mutant cells as independent variables. Categorical variables were incorporated into the regression model by indicator design matrix with curve 1 as baseline. The interaction terms are delimited by semicolons.
(XLSX)

Table S3 **Parameters associated with the tunneling probability.** Regression coefficients associated with the number of times tunneling is observed in 1,000 simulations for each combination of the 5 proliferation kinetic curves, 5 death selection curves, 4 death rates (0, 0.01, 0.1, 2), and 3 fitness values for *APC* ^+/−^CIN cells (0.5, 1.0, 2.0) and 3 fitness values for *APC* ^−/−^CIN cells (0.5, 1.0, 2.0). Significantly associated covariates are highlighted (p-value<0.05). Positive estimates indicate that an increasing tunneling rate is associated with the parameter. Negative estimates indicate that a decreasing tunneling rate is associated with the parameter. Associations between the frequency of tunneling and the effects of input parameters are assessed using linear regression. The linear regression (least square fitting) was performed with the number of times tunneling occurs as the dependent variable, and proliferation kinetic curves (categorical variables), death selection curves (categorical variables), the interaction between proliferation and death curves (categorical variables), death rate, relative fitness of mutant cells, interaction between proliferation curves and death rate (categorical variables), interaction between death selection and death rate (categorical variables), and three-party interaction between proliferation (categorical variables), death selection and death rate as independent variables. Categorical variables were incorporated into the regression model by indicator design matrix with curve 1 as baseline. The interaction terms are delimited by semicolons.
(XLSX)
