# Supplementary figures and images for: Patterns of Proliferative Activity in the Colonic Crypt Determine Crypt Stability and Rates of Somatic Evolution
Source: PLoS Comput Biol. 2013 Jun 13;9(6):e1003082. doi: 10.1371/journal.pcbi.1003082 (PMC3681728; doi:10.1371/journal.pcbi.1003082)

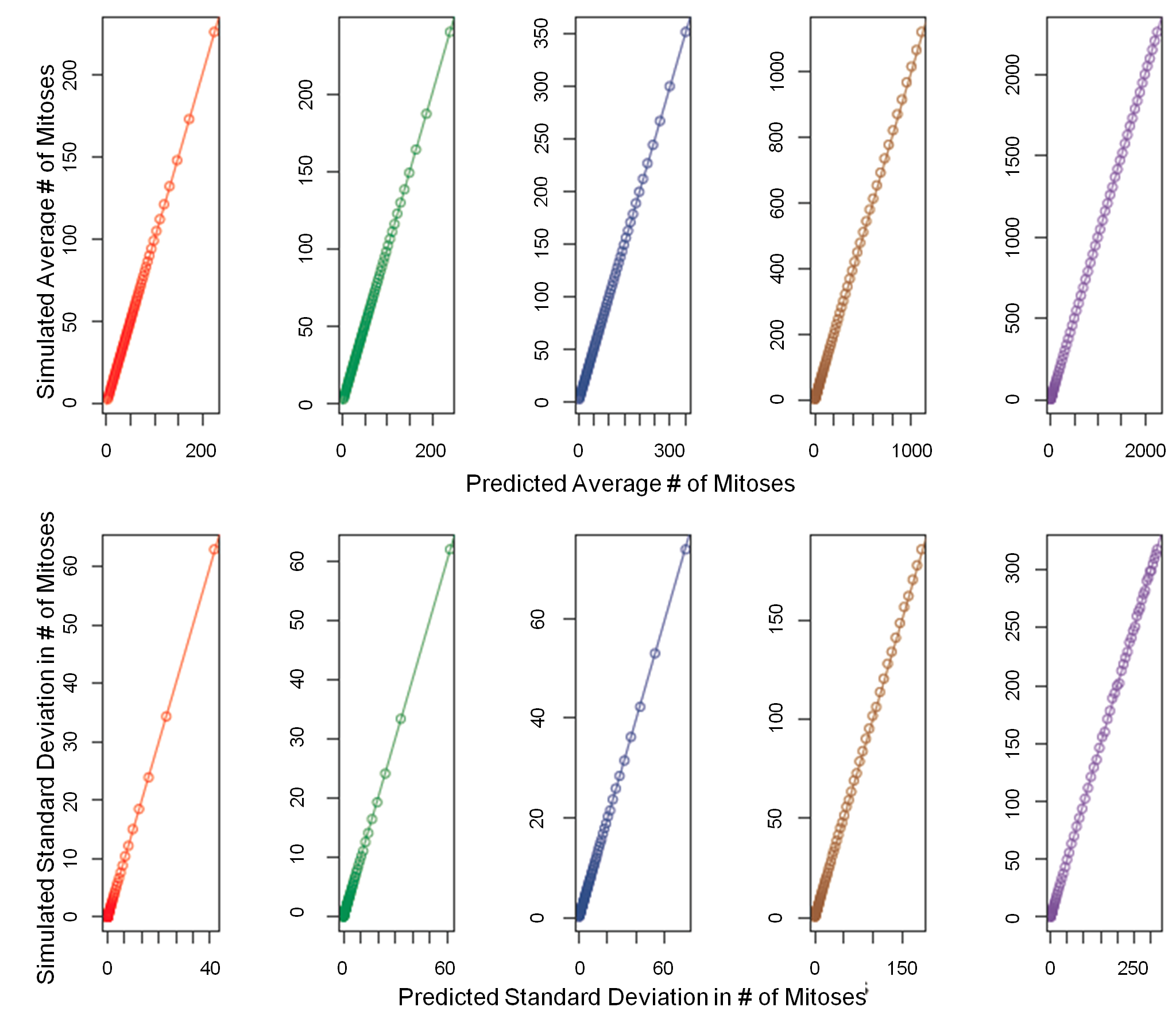

Supplement: Figure S1 — Concordance between results and analytical results from Markov chain in the absence of cell death. Top row: the average number of cell divisions needed for cells at positions 2–80 to exit the crypt column. Each dot represents a position (2–80) in crypt column. Bottom row: the variance associated with the number of cell divisions. Simulated results are based on 1,000 simulations. As expected simulated results and analytical results fall on a straight line. Color corresponds to the different proliferation curves in Fig. 1. (TIF) [file pcbi.1003082.s001.tif]

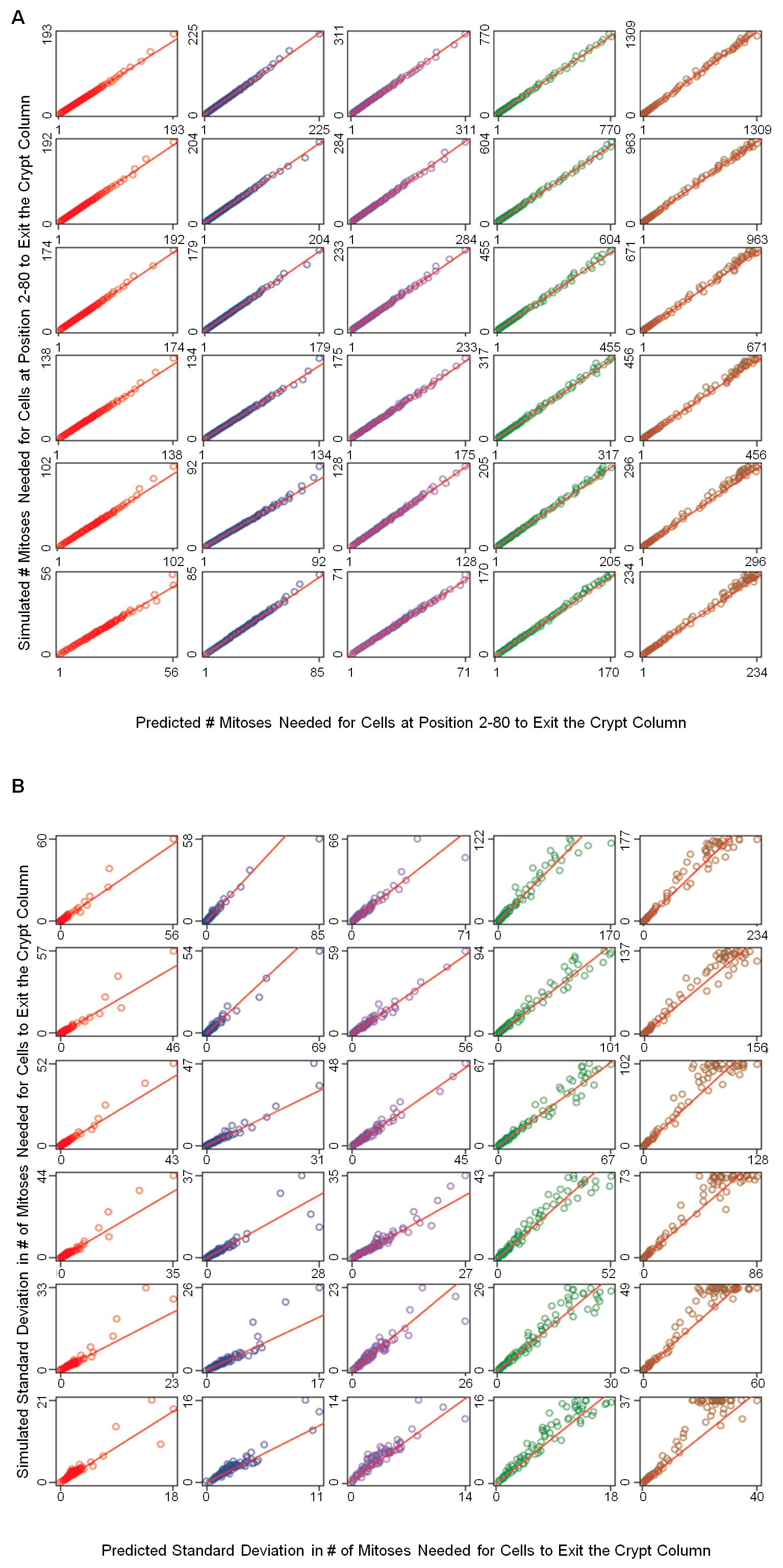

Supplement: Figure S2 — Concordance between simulated and analytical results from Markov chain in the presence of cell death. A: the average number of cell divisions needed for cells at positions 2–80 to exit the crypt column. Each row represents a particular death rate, λ, from top to bottom, λ = 0.1, 0.2, 0.4, 0.8, 1.6 and 3.2. B: the variance associated with the number of cell divisions for λ = 0.1, 0.2, 0.4, 0.8, 1.6 and 3.2. As expected simulated results and analytical results fall on a straight line. Color corresponds to the different proliferation curves in Fig. 1. Simulated results are based on 1,000 simulations. (TIF) [file pcbi.1003082.s002.tif]

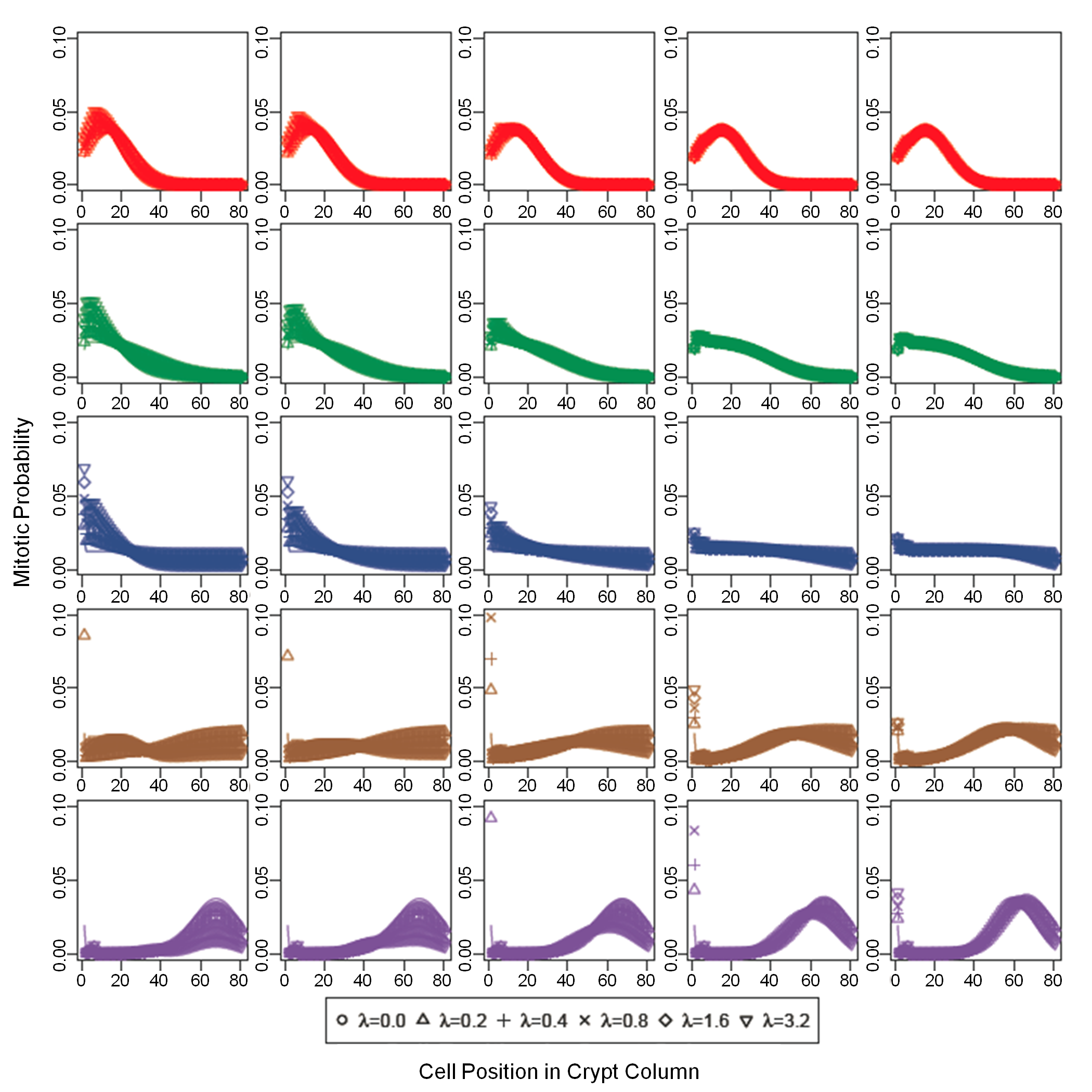

Supplement: Figure S3 — Overall proliferation curves for various combinations of proliferation, death curves and death rate. Each row represents a proliferation curves, curve 1 on the top and curve 5 at the bottom. Each column represents a particular death selection curve, death curve 1 on the left and death curve 5 on the right. Seven death rates (λ) are tested ranging from 0 to 3.2. Cell death and replenishing cell divisions results in deviations from the original proliferation curve (λ = 0). (TIF) [file pcbi.1003082.s003.tif]

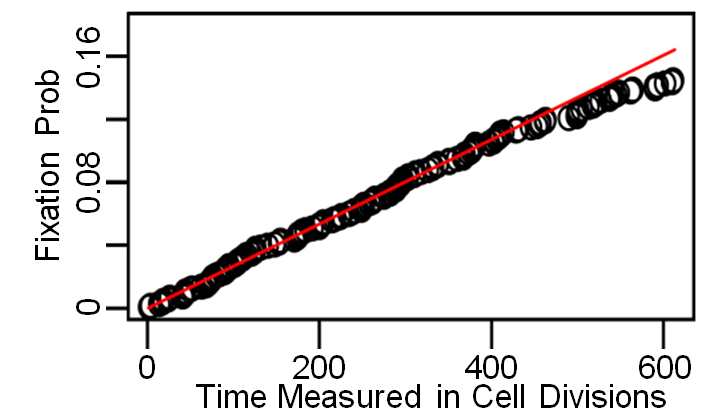

Supplement: Figure S4 — Concordance between simulated and analytical rates of somatic evolution. Comparison between the simulated somatic evolution rate and analytical approximated rate (Eq. 8), using u 0 = 0.01, proliferation kinetics curve 5, death curve 3 and death rate λ = 0.5 with 1,000 simulation runs. The red line indicates the analytically approximated results and black dots indicate the simulated results. The number of cell divisions is truncated at time 600 to compensate for the artificially high mutation rate (u 0 = 0.01) used to speed up the simulation. The cut of time at 600 is selected based on the number of cell divisions in a crypt column: 24 division/day × 365 days/year × 70 years = 613,200 divisions per lifetime; and to compensate for difference between u 0 used in the simulation and the actual value, u 0 = 10−7, such that we have 613,200/(0.01/10−7) = 6.13. This number of divisions is scaled up by a factor of 100 to demonstrate the robustness of Eq. 8. (TIF) [file pcbi.1003082.s004.tif]

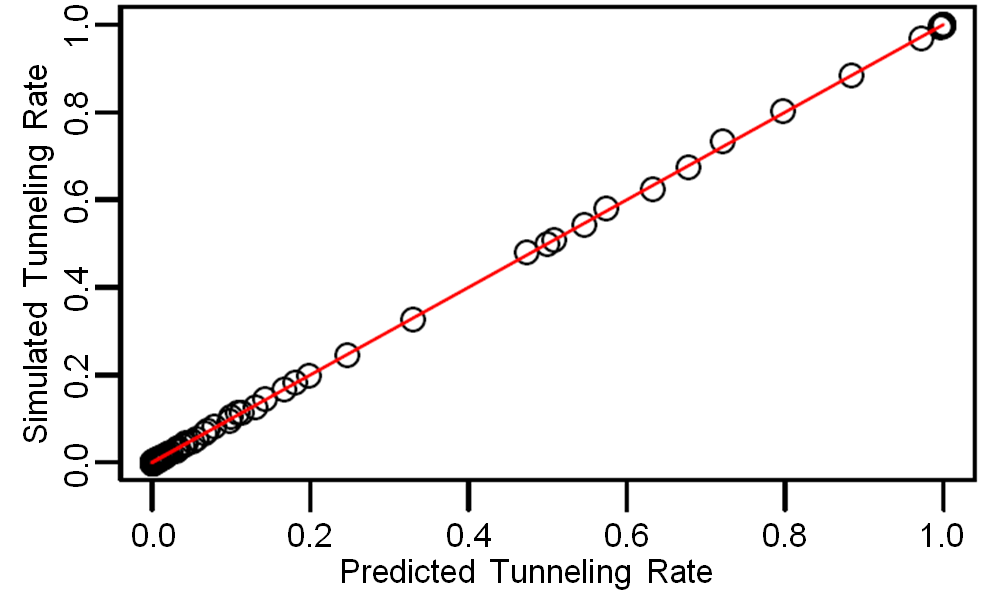

Supplement: Figure S5 — Tunneling probabilities are not sensitive to fitness variation in the absence of cell death. X-axis shows the calculated tunneling probabilities using Eq (14) which does not require relative fitness values as inputs. Y-axis shows the simulated tunneling probabilities. Simulated tunneling probabilities are simulated using linear systems of length = 10, … 100 with equal proliferation probability at each position, mutation rate u 3 = 0.001 …1.0 and relative fitness values of 1.0, 1.5 and 1.0 for APC+/−, APC+/−CIN and APC−/−CIN cells respectively. Simulated results are based on 1,000 simulations. (TIF) [file pcbi.1003082.s005.tif]
